# What support do caregivers of people with visual impairment receive and require? An exploratory study of UK healthcare and charity professionals’ perspectives

**DOI:** 10.1038/s41433-021-01821-6

**Published:** 2021-11-06

**Authors:** Jamie Enoch, Christine Dickinson, Ahalya Subramanian

**Affiliations:** 1 Division of Optometry and Visual Sciences, School of Health Sciences, City, University of London, London, UK; 2 Division of Pharmacy & Optometry, University of Manchester, Manchester, UK

**Keywords:** Health services, Eye diseases, Quality of life

## Abstract

**Background:**

Previous research has established that some informal caregivers (relatives/friends) of people with visual impairment (PVI) may require support themselves. However, there is limited understanding of how healthcare services and sight charities in the UK currently support caregivers. This study was therefore conducted to explore what support, information, and advice healthcare and charity professionals (HCCPs) currently provide for caregivers, and which additional support HCCPs would recommend in order to benefit caregivers.

**Methods:**

HCCPs filled out an online survey, distributed among UK-based professional bodies and charity partners. Of 104 individuals who consented to participate, 68 (65%) HCCPs completed the survey in September–November 2019. Participants responded to Likert-type questions about how they interact with and support caregivers of PVI. Thirty-eight (56%) participants provided responses to open-ended questions about improving support for caregivers; qualitative analysis was conducted using the Framework Method.

**Results:**

The survey showed that caregiver support activities most commonly undertaken related to onward signposting (90% (95% CI: 82–97%) of participants), or providing information about low vision aids and adaptations (85% (95% CI: 77–94%)), compared to activities focused on broader caregiver wellbeing. In open-ended responses, HCCPs highlighted the difficulties caregivers face in navigating an under-resourced and complex system. They recommended improving coordination and accessibility of information, as well as provision of emotional support and tangible assistance such as respite care and financial support.

**Conclusions:**

The study suggests that HCCPs perceive significant unmet needs among caregivers of PVI, and would welcome further resources, information, and training to support caregivers.

## Introduction

Around 2.5 million people in the UK [1], and an estimated 285 million people worldwide [2], are living with sight loss. Many individuals living with visual impairment (VI) will receive regular support from family members or friends, sometimes referred to as ‘informal caregivers’ [3]. Large cross-sectional studies in Canada [4] and the USA [5] show that people with visual impairment (PVI) use significantly more informal home care than those without VI.

Previous research has established that caregivers of adults and children with VI may experience stress, anxiety, and/or depression linked to their caregiving role [6–8], and therefore may be in need of support. Research suggests that the level of caregiver distress may be linked to factors such as comorbidities experienced by the person with VI [9], or their level of functional vision [10, 11].

Some of the relatively sparse literature involving caregivers of PVI has elicited the perspectives of health and care professionals about caregiver support needs and services. For example, a 2009 UK-based qualitative study involved in-depth interviews with several participant groups about dementia and visual impairment, including low vision professionals [12]. That study [12] found that low vision professionals were aware of the magnitude of the caregiving task faced by informal caregivers of people living with both dementia and VI, and recommended additional respite resources in order to reduce caregiver distress. Additionally, a qualitative study explored Australian vision rehabilitation professionals’ views on the benefits and drawbacks of involving family members and friends of PVI in group-based low vision rehabilitation programs [13].

These aforementioned studies have incorporated professionals’ perspectives on issues relevant to caregivers and PVI together. However, to the best of our knowledge, no study has specifically explored how professionals involved in the support of PVI interact with and support the informal caregivers of their patients, clients, or service users with VI. A study was therefore conducted in order to explore two interlinked research questions: firstly, what support, information, and advice healthcare and charity professionals (HCCPs) in the UK currently provide for caregivers of PVI; and secondly, which additional strategies HCCPs would recommend in order to enhance support for caregivers of PVI. (A separate study will consider the views of caregivers themselves, regarding how they could be better supported.)

## Methods

### Development of the survey

A literature search was initially conducted for studies exploring the experiences of health professionals (within and outside ophthalmology) working with informal caregivers. No studies were identified using validated questionnaires that were relevant to the present study’s research questions. Therefore, a survey was developed by the study authors, in consultation with an advisory group consisting of individuals with VI and caregivers.

The survey was designed in order to understand the views of HCCPs, regarding the type of support, referrals and advice they currently provide to caregivers of PVI; their thoughts on the quality of the support available for caregivers; and suggestions for improving support. The survey combined Likert-type scale questions and four open-ended questions. Information was also collected on participant demographics and characteristics of the participant’s service user base (e.g., age of most clients/patients). The full survey questions are shown in Appendix 1.

Next, the survey was piloted with three experienced eye care professionals, to check for face validity and readability of the instrument. Following their comments, a second iteration of the survey was developed. The survey received final approval from the City, University of London School of Health Sciences’ Optometry Research Ethics Committee (reference ETH1819-1338).

The final version of the questionnaire was then distributed online via Qualtrics in September–November 2019 and publicised by relevant UK-based professional bodies and charity partners (please see Acknowledgements). The survey was open to UK-based participants aged 18 or over who considered themselves to be in regular professional contact with visually impaired clients, service users, or patients. Written informed consent was obtained from all participants.

### Data analysis

#### Quantitative responses

Descriptive analysis of demographic information and responses to the Likert-type scale questions was undertaken. Where appropriate, chi-square tests of goodness-of-fit were used to explore whether one question response was chosen significantly more often than other responses. For the question asking participants to select activities conducted with caregivers, the Friedman test (two-sided) and post-hoc pairwise comparisons with Bonferroni’s correction were undertaken to establish if there were significant differences between the frequencies of the different activities. Statistical tests were conducted using SPSS, version 25.0 (SPSS Inc., Chicago, IL, USA).

#### Qualitative responses

To analyse the qualitative data afforded by the open-ended survey questions, we used the Framework Method [14], a systematic approach to qualitative data analysis. The matrix output generated through the Framework Method allows not only for the identification of broad patterns across the dataset but also close attention to individual cases. This comprehensiveness was considered valuable for our exploratory applied study, to ensure that potentially useful, innovative ideas and insights were included and retained, even where these did not fit neatly into the overarching categories.

Responses to each of the four open-ended questions were analysed in turn by the first author (JE). The first step was familiarisation with the dataset through several readings and making some initial analytical notes. Formal open coding was then used to classify relevant, meaningful elements of the dataset. Once codes were developed and refined, these were grouped into higher-order categories. The categories and constituent codes were then discussed and confirmed with the senior researchers on the study (AS and CD), to finalise an analytical framework. The software NVivo 12 Pro (QSR International, Melbourne, Australia) was used to sort the qualitative data into a framework matrix, with a column per code and a row per participant. Reviewing the framework matrix as a whole allowed for the development of themes, to encapsulate meaningful patterns in the responses.

## Results

Out of 104 individuals who read the participant information online and consented to participate, 68 (65%) completed the survey.

### Characteristics and professional profile of participants

Table 1 displays characteristics of the 68 HCCPs who participated in the study, as well as attributes of the main service user groups with whom the HCCPs reported working. As shown in Table 1, optometrists were the most represented profession among participants, accounting for 29% (*N* = 20) of responses. Participants had varying levels of experience; 37% of participants had worked for 1–5 years with PVI, while 28% had worked with PVI for over 20 years. Generally, participants were focused on working with older adults, with 84% of participants working with service users over 85 while only 28% were working with service users under 19. Participants were significantly more likely to say that most or all of their service users were older adults over 65 than not (*χ*^2^ (1) = 15.06, *P* = < 0.001). Age-related macular degeneration (AMD) was the most frequently selected cause of visual impairment among service users, selected by 93% of participants. In contrast, 37% of participants reported eye conditions commonly causing visual impairment in children, such as cerebral visual impairment and childhood ocular blindness, as common causes of VI among their service users.

**Table 1:** Characteristics of participants (*N* = 68) and their service users.

Characteristic	*N*	*%*
**Profession**		
Optometrist	20	29
Charity professional	18	26
Eye care liaison officer (ECLO) or sight loss adviser	10	15
Dispensing optician	7	10
Rehabilitation officer/worker (Visual Impairment)	5	7
Other professional	3	4
Orthoptist	2	3
Nurse	1	1
Ophthalmologist	1	1
Teacher of the visually impaired	1	1
**Location of participant’s organisation within the UK**		
England	50	74
Northern Ireland	5	7
Scotland	4	6
Wales	1	1
Did not provide the location (*this question was optional*)	8	12
**Time spent working with PVI**		
Less than 1 year	4	6
1–5 years	25	37
6–10 years	10	15
11–15 years	5	7
16–20 years	5	7
Over 20 years	19	28
Age of service users with whom participants work*		
Under 19	19	28
19–24	24	35
25–34	26	38
35–44	27	40
45–54	28	41
55–64	45	66
65–-74	56	82
75–84	60	88
Over 85	57	84
**Are the service users you work with all or predominantly older adults?**		
Yes	50	74
No	18	26
Main causes of service users’ visual impairment*		
Age-related macular degeneration	63	93
Cataracts	38	56
Cerebral visual impairment	25	37
Childhood ocular blindness	25	37
Diabetic retinopathy	43	63
Eye injuries or infections	17	25
Glaucoma	44	65
Neurological disease (e.g., visual impairment after stroke or trauma)	41	60
Rare inherited eye diseases (e.g., Retinitis pigmentosa, Leber congenital amaurosis, Stargardt disease)	41	60
Other	5	7
Level of service users’ visual impairment*		
Mild vision loss e.g., still eligible to drive a car	52	76
Moderate visual impairment e.g., requiring optical or electronic magnification to aid vision	63	93
Little functional vision, relying on auditory and tactile information	35	51
(Registered) sight impaired, or “partially sighted"	53	78
(Registered) severely sight impaired, or “blind”	47	69
No light perception	22	32
**Do your service users (or their caregivers) ever mention or show signs of Charles Bonnet Syndrome?**		
Always	2	3
Frequently	33	49
Sometimes	29	43
Never/Almost never	4	6
**How often do service users you work with have another chronic condition which significantly impacts their health?**		
Always	2	3
Frequently	50	74
Sometimes	16	24
Never/Almost never	0	0
Don’t know	0	0
**Could the service users you work with generally manage independently at home, without external help/support?**		
Always	1	1
Frequently	8	12
Sometimes	49	72
Never/Almost never	10	15
Don’t know	0	0

^*^For these questions, participants could select more than one response.

### Quantitative survey responses

Data from the Likert-type scale questions about HCCPs’ perceptions and practices are shown in Table 2a, b.

**Table 2:** (a, b) Results from Likert-type scale questions on the survey regarding views on caregiver support.

	*N* (%)				
	Always	Frequently	Sometimes	Never/almost never	Don’t know*
How often do you interact with caregivers (e.g., family members, friends, formal carers) of the service users you work with?	9 (13)	39 (57)	19 (28)	1 (1)	
Do you generally check whether a person accompanying your service users is their “caregiver" (i.e., is regularly involved in their care and support at home)?	34 (50)	18 (26)	9 (13)	7 (10)	
Do caregivers you meet in your role appear to be struggling to cope?	3 (4)	6 (9)	47 (69)	7 (10)	5 (7)
Are the caregivers you meet generally vulnerable or in a state of poor health?	1 (1)	6 (9)	41 (60)	17 (25)	3 (4)
Do you feel confident providing advice, support and/or information to caregivers you meet?	24 (35)	22 (32)	21 (31)	1 (1)	0 (0)
Do you generally think that the caregivers you meet in your role would benefit from additional practical or emotional support?	16 (24)	36 (53)	13 (19)	1 (1)	2 (3)
Do you think that caregivers require more support to look after the service user’s vision and eye health specifically (e.g., managing medications, applying eye drops etc.)?	7 (10)	20 (29)	34 (50)	6 (9)	1 (1)
Would the caregivers you interact with benefit from more generic carer support services (e.g., counselling, respite care, financial/legal advice)?	7 (10)	31 (46)	24 (35)	1 (1)	5 (7)
When caregivers are clearly in need of extra support, are you clear about where to refer them?	21 (31)	20 (29)	21 (31)	6 (9)	
Are you generally confident that caregivers have timely access to the support you recommend?	3 (4)	16 (24)	37 (54)	3 (4)	9 (13)

Question	*N*	%
**Have you ever raised a safeguarding alert out of concern for the caregiver of a service user?**		
Yes	4	6
No	64	94
**Which of these general activities, if any, do you undertake with caregivers of your service users?**^**#**^		
Provide advice to the caregiver about housing, transport, education, benefits and/or employment	26	38
Provide information to the caregiver about the patient/client’s vision loss and prognosis	56	82
Provide advice to caregivers on supporting the patient/client’s eye health (e.g., medication management, applying drops)	46	68
Discuss aids or strategies that may reduce caregiver burden/effort with daily activities (e.g., low vision aids, other assistive technologies, home adaptations)	58	85
Discuss the emotional aspects of caregiving	30	44
Signpost caregiver to other sources of help (including sight loss and carer support charities)	61	90
Refer caregiver to social services	26	38
Provide advice to caregivers on looking after their own physical and/or mental health	23	34
Provide counselling (or other formal psychological therapy) to caregiver	6	9
**If more information/training regarding caregiver support was available to professionals, would you be interested?**		
Yes	55	81
Yes, but too busy	8	12
No, I feel I know enough already	3	4
Not interested/not relevant to me	2	3
**Do you feel that the overall quality of the support system for caregivers of your service users is generally:**		
Excellent	2	3
Good	11	16
Adequate	25	37
Poor	20	29
Don’t know	10	15

*Where cells are blanked, this denotes that the option was not available.

^#^For this question, participants could select all responses that applied.

There were significant differences in the kind of support activities HCCPs reported undertaking with caregivers (Friedman’s *χ*^2^ (8) = 186.87, *P* < 0.001). The support activities undertaken by participating HCCPs were most commonly practical and/or related to vision; these included signposting of caregivers to other sources of help such as sight loss or caregiver support charities (undertaken by 90% (95% CI: 82–97%) of participants), as well as discussion of low vision aids and adaptations (85% (95% CI: 77–94%)) and information provision (82% (95% CI: 73–92%)). In comparison, activities focused on broader caregiver wellbeing (such as discussing the emotional aspects of caregiving, or advice to caregivers about looking after their own health) were undertaken significantly less commonly (Bonferroni-adjusted pairwise comparisons using Wilcoxon signed-rank tests, between each of the three practical activities and the two wellbeing activities: all *P* < 0.001).

### Qualitative findings

In addition to the quantitative data presented above, 38 participants (56%) responded to some or all of the four open-ended questions. Responses to each of the four questions were analysed in turn using the Framework Method [14]. The four questions generated responses that overlapped in parts, while also raising distinct concerns and suggestions for improving services for caregivers of PVI.

Firstly, HCCPs discerned a difficulty for caregivers in understanding the “hidden” but wide-ranging impacts of vision loss (Table 3). Participating HCCP’s also discussed the variety of activities affected by VI, which makes it difficult to formally recognise or quantify what caregiving may entail. HCCP’s referred to the emotional impacts for both the PVI and caregiver, often compounded by a shared uncertainty about the future. They also discussed the difficulty of the PVI and caregiver understanding each other’s feelings and frustrations, for example, the balance of the caregiver providing support without impinging upon the independence or privacy of the PVI.

**Table 3:** “In your view, what makes caring for someone with a visual impairment different to caring for someone with another chronic health condition?” (38 responses).

Theme derived from Framework Analysis	Description	Illustrative quotation(s)
The difficulty of understanding visual impairment and what the person with visual impairment (PVI) sees	Participants frequently stated that it was hard for caregivers to fully understand the impact of sight loss on the PVI. Several participants referred to visual impairment being an invisible condition.	“Vision loss can’t be seen by the caregiver” (P27). “[VI is] a hidden disability” (P14, P46, P49) “[VI is] not obvious” (P5, P31) “Simulation specs are useful” (P62) for allowing caregivers to see what the PVI sees.
Specific practical challenges of caregiving	Participants described the way in which sight loss can impact a significant variety of daily tasks with which caregivers may need to provide support. Providing informal care may therefore pervade all aspects of daily life. This may make it difficult to plan for where and when support for caregivers may be required.	“Sight loss impacts on most aspects of daily living which means a range of disability can be very wide.” (P50) “Caring for someone with a visual impairment can be a 24 hours job.” (P21) “It’s more difficult to formally recognise/quantify the level of support because it [caregiving] is often lots of very small unplanned "events" dispersed through the day, rather than a very specific single event that can be planned on a regular basis.” (P11)
Emotional impact on the caregiver-PVI together	Both PVI and caregivers may face isolation, loneliness and frustration. The mental health impacts of VI may thus create an additional challenge for caregivers. The uncertainty about the PVI’s prognosis means caregivers may have to constantly revise their expectations for the future.	“Emotional impact of sight loss can be very severe for all involved (knock-on effect).” (P14) “Frustration with the change to their own hopes and ambitions.” (P46) “VI increases the risk of depression for the patient which would make caregiver role more challenging, as [it’s] not just visual problems to contend with.” (P63) “Parents of children with progressive diseases have to adapt to new reality’ every year or two, potentially with implications on hopes and aspirations for their child.” (P41).
Potential relationship strain between the caregiver and PVI	Several HCCP’s highlighted the dilemma experienced by caregivers between providing assistance and preserving the independence of the PVI. There may be mutual frustrations and divergences in the PVI’s and caregiver’s views on what may be helpful. Responses pointed to the balance required between the caregiver’s active involvement in the PVI’s life, and respect for the PVI’s autonomy and privacy.	“The patient is still capable and desires independence but still needs sometimes resented support.” (P52) “It can be hard for the caregiver to understand the bereavement felt… Caregivers often look for solutions and are frustrated if the client doesn’t want the gadgets etc available.” (P32) “Often doing the right thing such as tidying up can be counterproductive to the person with VI, causing friction.” (P49) “VI… may impact on the privacy of patient due to caregiver possible need to read correspondence, [which] risks affecting [the] relationship” (P63).

Themes generated from the Framework Analysis revealed perceived gaps or shortcomings in the provision of support for caregivers, including the need for improved emotional support for caregivers, improved information and advice about living with VI, respite care options, financial and benefits advice, and support with transport (Table 4). The HCCPs also drew attention to more systemic issues, such as the difficulties navigating and accessing available services, and the overstretched and underfunded nature of low vision services, which has created shortcomings in support available to PVI, let alone caregivers.

**Table 4:** “What do you perceive to be the most serious gap or shortcoming in the provision of support for caregivers of people with a visual impairment?” (36 responses).

Theme derived from Framework Analysis	Description	Illustrative quotation(s)
Gaps in emotional support	Emotional support is important for the caregiver in its own right, and can also help support the wellbeing of the PVI. Caregivers may be under strain but may not reach out for help, especially if they do not recognise their tasks as caregiving.	“If they [*caregivers*] are not coping, then it can impact on the support that they provide to the individual with the sight impairment.” (P24) “There is a view that losing your sight is part of old age and also to care for someone in old age is inevitable.” (P16) “Mindfulness for carers or stress-related courses.” (P65)
Unmet needs for information and advice about living with VI	Professionals highlighted the difficulty caregivers may have in fully grasping the extent or implications of the PVI’s vision impairment, and some HCCP’s suggested advice for caregivers to improve their understanding and awareness of conditions associated with VI. Many HCCP’s recommended easy access for caregivers to up-to-date contact details of local support organisations, as well as clear advice from healthcare professionals.	“Watching your loved one read the top line of a chart does not give a clear indication as to the difficulties they face.” (P34) “[We need] the time to explain how to help, what’s the condition and how it affects the patient so that they [caregivers] know best what to expect.” (P52) “More information is required in the public domain for people who suffer from Charles Bonnet Syndrome, quite often people won’t mention hallucinations as they are frightened that they have a serious mental health condition.” (P66) “Good quality advice provision- not just signposting.” (P40)
Respite for caregivers	The need for respite - allowing caregivers to take breaks - was mentioned by many participants. Respite was considered not only as a means of giving caregivers time for themselves, or to maintain their broader social life, but also an important factor in maintaining good relationships between the caregiver and PVI.	“A service that can enable carers to take some time off for themselves e.g., a part-time carer or a service [where] people with a visual impairment can go for a few hours so the carer can have a rest or time for themselves.” (P21) “Someone to provide respite so they can socially connect with the world around them.” (P65) “Understanding time apart is healthy and they don’t have to do it all.” (P44)
Gaps in practical, tangible support	There was a perceived need for better benefit advice and financial support for caregivers. Transport was also discussed as a crucial instrumental barrier or enabler for PVI and caregivers to benefit from the support services available.	“Lack of paid-for resources for respite or carer break[s]. Patients not having enough resources to allow them to spend attendance allowance/PIP [Personal Independence Payment] on specific disability support.” (P50) “A realistic benefit that reflects what the caregiver saves the Government financially” (P14). “[There is a gap in] transport and support for people with a VI to access social and need-based activities, therefore meaning the carer is required for transport.” (P49)
Navigating and accessing services	Participants mentioned finding the way to the appropriate service or information as a key obstacle for caregivers. HCCP’s may have limited awareness of the services available or suitable for caregivers. Indeed, participants also drew attention to the lack of identification or recognition of caregivers as a stakeholder group.	“Once in touch with relevant agencies, more often than not, issues can be addressed but if a carer is struggling, finding time to find the help can be difficult.” (P30) “The family [is] left to cope alone if not aware of services available.” (P32) “I think we have got much better as low vision professionals at referring people with visual impairment to counselling services, but in my experience, this hasn’t extended to caregivers.” (P41) “A separate provision is required dedicated to the carer.” (P59)
Services being stretched and underfunded	Many participants referred to the services available for PVI being stretched, thereby increasing the difficulty of accessing support for PVI and caregivers alike. This could create a vicious circle, in which a scarcity of formal support for the PVI increases demands on caregivers, who in turn have limited access to adequate support themselves.	“A lack of funding/services. The burden is put on family/friends.” (P27) “The limited and long wait for home assessment and mobility advice services in some areas.” (P18) “The reduction in local social service/low vision assessment support.” (P46) “Very long wait for the VI person to be seen by the VI rehab team in their home.” (P48)

The two final open-ended questions concerned the additional training, information and resources HCCPs might find useful to support caregivers of their service users, and any other thoughts about improving support for caregivers, respectively. As both questions concerned suggestions for service improvement, the data were analysed in combination (Table 5). In response to the shortcomings perceived by participating HCCPs (detailed in Table 4), participants recommended improving the accessibility of information, both on- and off-line, and access to up-to-date details for signposting and referring caregivers to both local and national organisations. Regarding training, participants referred to the importance of raising professional awareness of caregivers’ issues by hearing directly from caregivers, and upskilling professionals to provide appropriate emotional support to caregivers. Participants advocated increased and more equitable funding for low vision and rehabilitation services, which can support caregivers as well as PVI. They also highlighted the importance of recognising that caregivers may need flexible support adapted to the complexity of their living and caring situations.

**Table 5:** Improving the support provision for caregivers.

Theme derived from Framework Analysis	Description	Illustrative quotation(s)
Improving the accessibility of information to help guide and signpost caregivers	Some participants commented that they had all the information they needed. However, many emphasised the importance of information about support services at the local level. In particular, HCCP’s recommended accessible, consolidated information readily available offline as well as online; for example, one participant suggested a more defined pathway and “who’s who” to help the caregiver to navigate the care and support system, and to know whom to approach at what stage. It was recognised that keeping information up-to-date was a challenge, as individual and organisational responsibilities are continually changing. Overall, HCCP’s would benefit from quick access to up-to-date contact details of relevant local and national organisations to whom they can signpost or formally refer caregivers, as well as to seek information themselves.	“I have the information I need - leaflets, information about other agencies.” (P31) “Information about local services for caregivers; leaflets/booklets that could be given to caregivers; information about any groups or helplines that might be useful.” (P28) “There is plenty of support available but it is generally online and confined to disease-specific groups.” (P34) “Services that are available are generally unknown to the public. If the required info was more readily available, I would feel more confident in passing it on.” (P43) “One place to receive all the up to date, current information you need at that point in time.” (P14) “Easy access to contact details of local contact support organisations including benefits advice. Recently there have been changes in local organisations doing visual impairment support.” (P18)
Additional learning and training for professionals	Participants expressed a wish for generic training in working with caregivers and raising awareness about caregiver support across the low vision workforce. There were also specific training avenues suggested, such as emotional support to upskill professionals in engaging in sensitive conversations with caregivers. Participants also referred to the importance of listening to and better understanding caregivers’ lived experiences, in order to inform their professional practice.	“Improving awareness that [caregiver support] is an area requiring additional input… It starts with training, it is not something we think about.” (P54) “More training to provide emotional support that will encourage people to open up and talk about their own feelings, but will enable a conversation that will encourage to access support through their GP, Counsellor, etc.” (P24) “Hearing from a caregiver what their day involves, the highs and lows, so [that] as professionals we learn from them.” (P44)
Funding to improve how services structure and deliver caregiver support	Participants discussed inconsistencies in the caregiver support infrastructure in the UK. Some participants saw support for caregivers as underfunded, inequitable, and - when available – rarely extending beyond the period when a condition is diagnosed or a PVI is certified as visually impaired. The under-resourcing of services prevents timely access to low vision services that can support both the PVI and the caregiver.	“This varies so much, [it’s] a postcode lottery. Cuts in statutory services leaves caregivers stressed and often ill themselves. Support not just at diagnosis or registration - it needs to be ongoing.” (P32) “Often rehab staff are insufficiently resourced so timely intervention for both the individual and the carer is often lacking.” (P30)
Adapting services to the complexity of caregivers’ living situations	Even where support for caregivers is of good quality, caregivers may be unable to benefit due to constraints imposed by their caregiving role. This suggests that for caregivers to benefit appropriately, support services need to be flexible and adapted to the complexity of caregivers’ living situations.	“We have a very good carer’s support service, the problem is that a lot of people cannot attend due to their caring role. Maybe some kind of app or online carer’s support where they can discuss how they are feeling or have a chat without physically getting out” (P65).
The importance of public services recognising caregivers of PVI	Participants recommended inculcating awareness and recognition of caregivers’ role across services.	“Recognition for what caregivers do” (P42). “Awareness from government agencies of the impact on carers of coping with someone with low vision.” (P46)

Themes and quotations in this table were generated from two open-ended survey questions: “What kind of additional information/training/resources to better support the caregivers of your service users would be useful to you (if any)?” (30 responses) and “If you have any other thoughts about improving support for caregivers, please briefly note them here” (17 responses).

## Discussion

The results from this exploratory study suggest that HCCPs would be receptive to further training and information on supporting caregivers, although our sample was likely to be more interested in this issue than non-responders.

Our findings suggest that HCCPs more commonly provide ‘informational support’ [15] (signposting or advice provision) for caregivers, than ‘emotional support’ activities. This may not be particularly surprising given that emotional support activities are likely to be more intensive, specialist, and not necessarily built into HCCPs’ professional roles and responsibilities. Indeed, people directly affected by VI or eye disease have discussed a lack of empathy from health professionals and an unmet need for emotional support [16, 17] and participants in the present study suggested such issues may also affect caregivers. Many participants mentioned how distress experienced by one member of the dyad may in turn affect the other, a phenomenon that has been demonstrated among PVI and their spouses in the USA [18]. No studies, to the authors’ knowledge, have directly assessed psychological distress among caregivers of PVI in the UK; however, caregivers of people with neovascular AMD have been found to show high levels of burden and distress on the Caregiver Reaction Assessment, even when approximately half of the patients had good visual acuity (of at least 6/12 Snellen) [19]. In the present study, there was a strong awareness of the psychological impacts of vision loss on PVI and caregivers, and HCCPs believed they could benefit from more time with PVI and caregivers to set expectations and provide meaningful advice. Many participating HCCPs also highlighted the potential value of, and their openness to, emotional support training, potentially in dialogue or collaboration with caregivers themselves.

Alongside emotional support, most participating HCCPs advocated additional practical support for caregivers; their ideas of the form this support could take were manifold, including a particular focus on respite care. Although no studies have focused wholly on respite care for caregivers of PVI, respite care more generally has been shown in randomised trials to moderately reduce caregiver distress [20], and qualitatively to yield concrete, often highly personalised psychological benefits for the caregiver [21, 22]. Many HCCPs also expressed the view that caregivers with whom they interact are struggling financially, and that current levels of financial support for caregivers are not sufficient and do not recognise the economic contribution of the caregiver. Indeed, informal caregivers, in general, save the UK government £132 billion annually [23]. More specifically, a recent Fight for Sight report estimates that informal care from family and friends of people living with sight loss amounts to an annual £8.5 billion (with the caveat that little is known about the financial impact on caregivers themselves, and so the real figure may be higher) [1].

Alongside suggestions for additional support resources or services for caregivers, there was awareness of the scarcity of existing resources for low vision assessment and rehabilitation for the PVI which itself may increase strain on caregivers. This underfunding also suggests it would be a challenge for existing services to expand to cover caregivers in a climate of increasing demand and limited funding. This was reflected in participants’ concerns that caregivers are not receiving timely and sustained access to support. Indeed, pressure on UK eye care services is predicted to increase in the coming decades as the number of people living with diseases like AMD, glaucoma, cataract and diabetic retinopathy increases [24]. This pressure – compounded by the COVID-19 pandemic [25] - is coupled with the decline in UK statutory health and social care services supporting PVI that has taken place over the last decade [26–28]. As such, experiences of caregivers’ access to support may be highly uneven and inequitable, described by one participant as the “postcode lottery”. Indeed, this is an observable phenomenon in other areas of eye health, for example in the variation documented across UK vision rehabilitation services [29]. Participants’ awareness of these pressures on low vision services suggests that although training and upskilling HCCPs to support caregivers could be helpful, there are fundamentally many systemic barriers to supporting caregivers. Considering the Capability, Opportunity, and Motivation Model of Behaviour (COM-B model) [30], an influential model of behaviour change, training could improve the ‘capability’ of HCCPs to work with caregivers; yet it would not necessarily address ‘opportunity’ barriers such as time constraints, which may leave little time for meaningful discussions with caregivers.

A majority of participants generally felt confident providing advice, support and information to caregivers. However, concerns about the sometimes disparate and inaccessible nature of information, and the complex system caregivers may need to navigate to reach appropriate support, were notable and reflect concerns expressed by PVI themselves [16]. Suggestions of consolidating information in one up-to-date ‘one-stop-shop’-style resource, and better clarification of referral pathways in order to facilitate caregivers’ access to the appropriate agency or service both at the local and national level, could potentially address these concerns. The benefits of ‘one-stop-shop' gateway-style models for information and advice provision have been explored in other areas, such as for people with autism spectrum disorder [31]. However, in a climate of under-resourced services, there may be uncertainties regarding how far these gateway services can guarantee substantive support at the end-stage of the pathway.

This was the first study to explore how UK healthcare and charity professionals work, interact with and support the caregivers of their clients, patients or service users with VI. As a small, exploratory study, there are a number of limitations. Firstly, there was inevitable selection bias, with participating HCCPs likely to be those more interested or involved in improving caregiver support. The survey was disseminated through charity partners and professional bodies, and there were differences in reach and uptake by different professional groups. For example, only one response was received from an ophthalmologist, compared with twenty responses from optometrists. Furthermore, we have no demographic data regarding non-responders, individuals who may have read the advert and participant information for the survey but chose not to participate. As such, the results have limited generalisability across the whole range of HCCPs working with visually impaired service users and clients. A further limitation is that our survey instrument was not a formally validated questionnaire, and there was limited scientific literature to guide its development. Although we consulted with members of the study’s patient and public advisory group and a number of health professionals to refine the instrument, we did not collect data on reliability, such as test-retest reliability. The Likert-type survey responses meant that participants were asked to, somewhat artificially, homogenise their varied professional experiences. For example, when asked if caregivers were struggling to cope, most participants (69%) responded “sometimes”, likely reflecting the broad range of conditions in which they may see caregivers and PVI, across a spectrum of ages, relationships, and health states. This, therefore, limits the granularity and precision of the survey data, although the collection of qualitative data helped to nuance findings from the quantitative section of the survey.

To conclude, our study suggests that HCCPs already tend to signpost caregivers to other services, and frequently provide caregivers with information and advice about coping with low vision. However, the findings suggest that professionals clearly perceive gaps in emotional support provision and tangible resources for caregivers, such as financial support and respite care. Several participants also discussed the manifold inequalities in access to and resourcing of caregiver support. In particular, participants expressed concerns around onward referral pathways and the caregiver’s timely access to appropriate support services. HCCPs highlighted the difficulties caregivers face in navigating an under-resourced and complex system, and the importance of better coordinating and streamlining information provision and the support pathway for caregivers. Participants were in favour of further resources, training and information to support caregivers. The co-design of an improved support service for caregivers, involving HCCPs and caregivers of PVI working together, could be a valuable avenue for future research.

### Summary Table

#### What was known before

- Previous research has established that some informal caregivers (e.g., relatives or friends) of people with visual impairment (PVI) may experience stress and require support themselves.
- A small number of studies demonstrate that healthcare and charity professionals (HCCPs) working with PVI seek to be inclusive of their clients’ relatives and friends.
- To date, no study has specifically explored how professionals involved in the support of PVI interact with and support the informal caregivers of their patients or clients with VI.

#### What this study adds

- Many participating HCCPs already provide certain forms of support to caregivers of PVI, especially in terms of practical low vision advice or onward signposting.
- Results from our exploratory study suggest that HCCPs would be receptive to further training and information on supporting caregivers both practically and emotionally.
- Our qualitative findings suggest that systemic pressures and stretched low vision services for PVI constrain the support that HCCPs can provide for the caregivers of PVI.

## Supplementary information

Appendix 1: Copy of survey instrument
