# Supplementary material for: What support do caregivers of people with visual impairment receive and require? An exploratory study of UK healthcare and charity professionals’ perspectives
Source: Eye (Lond). 2021 Nov 6;36(11):2179–87. doi: 10.1038/s41433-021-01821-6 (PMC8572072; doi:10.1038/s41433-021-01821-6)
Supplement: Supplementary file 1 — Appendix 1: Copy of survey instrument [file 41433_2021_1821_MOESM1_ESM.docx]

# Appendix 1: Copy of survey instrument

**Introduction**

Thank you for choosing to participate in this survey. The survey aims to explore how health, social care and charity professionals engage with the caregivers of their visually impaired patients/clients.

We define a caregiver in this survey as anyone regularly providing help or support for a visually impaired person, such as relatives, friends or neighbours. Therefore the term caregiver not only applies to paid carers but also to people who may provide more informal, everyday help or support to a visually impaired person.

1. **What is your professional role?**

Ophthalmologist

Optometrist

Orthoptist

Nurse

Eye care liaison officer (ECLO)/Sight loss adviser

Other doctor (please specify):

Rehabilitation officer/worker (Visual Impairment)

Psychologist, psychotherapist or counsellor

Physical, occupational, or speech and language therapist

Other health professional (please specify):

Social worker, caseworker, or other social care professional

Teacher of the visually impaired

Charity professional

Volunteer with a local organisation

Other professional (please specify):

1. **Please provide the first part of your organisation’s postcode (e.g. EC1) [*optional*]:**
2. **For approximately how many years of your career have you worked with patients, clients and/or service users with visual impairment?**

Less than 1 year

1 - 5 years

6 – 10 years

11 – 15 years

16 – 20 years

Over 20 years

1. **Are the service users you work with all or predominantly (please select all that apply):**

Under 19

19 – 24

25 – 34

35 – 44

45 – 54

55 – 64

65 – 74

75 – 84

Over 85

1. **Are the service users you work with all or predominantly older adults (i.e. aged 65 or over)?**

Yes

No

1. **Which are the main causes of visual impairment of the service users you work with (please select all that apply)?**

Age-related macular degeneration (AMD)

Cataracts

Cerebral visual impairment

Childhood ocular blindness

Diabetic Retinopathy

Eye injuries or infections

Glaucoma

Neurological diseases (e.g. visual impairment after stroke or trauma)

Rare inherited eye diseases (e.g. Retinitis pigmentosa, Leber congenital amaurosis, Stargardt disease)

Other - please specify: _________________

1. **How would you describe the vision of the service users you work with (please select all that apply)?**

No light perception

Little functional vision, relying on auditory and tactile information

Moderate visual impairment – e.g. requiring optical or electronic magnification to aid vision

Mild vision loss – e.g. still eligible to drive a car

(Registered) sight impaired, or “partially sighted”

(Registered) severely sight impaired, or “blind”

1. **Do your service users (or their caregivers) ever mention or show signs of Charles Bonnet Syndrome**^[[1]](#footnote-1)^**?**

Always

Frequently

Sometimes

Never/almost never

1. **How often do service users you work with have another chronic condition which significantly impacts their health?**

Always

Frequently

Sometimes

Never/almost never

Don’t know

1. **Could the service users you work with generally manage independently at home, without external help and support?**

Always

Frequently

Sometimes

Never/almost never

Don’t know

1. **How often do you interact with caregivers (e.g. family members, friends, formal carers) of the service users you work with?**

*[Interacting could be meaningfully involving the caregiver in discussions, for example]*

Always

Frequently

Sometimes

Never/almost never

1. **Do you generally check whether a person accompanying your service users is their ‘caregiver’ (i.e. is regularly involved in their care and support at home)?**

Always

Frequently

Sometimes

Never/almost never

1. **Do caregivers you meet in your role appear to be struggling to cope?**

Always

Frequently

Sometimes

Never/almost never

Don’t know

1. **Are the caregivers you meet generally vulnerable**^[[2]](#footnote-2)^ **or in a state of poor health?**

Always

Frequently

Sometimes

Never/almost never

Don’t know

1. **Do you feel confident providing advice, support and/or information to caregivers you meet?**

Always

Frequently

Sometimes

Never/almost never

1. **Do you generally think that the caregivers you meet in your role would benefit from additional practical or emotional support?**

Always

Frequently

Sometimes

Never/almost never

Don’t know

1. **Do you think that caregivers require more support to look after the service user’s vision and eye health specifically (e.g. managing medications or applying eye drops)?**

Always

Frequently

Sometimes

Never/almost never

Don’t know

1. **Would the caregivers you interact with benefit from more generic carer support services (e.g. counselling, respite care, financial/legal advice)?**

Always

Frequently

Sometimes

Never/almost never

Don’t know

1. **When caregivers are clearly in need of extra support, are you clear about where to refer them?**

Always

Frequently

Sometimes

Never/almost never

1. **Which of these general activities, if any, do you undertake with caregivers of your service users? (Please select all that apply.)**

|  | **Yes** | **No** |
| --- | --- | --- |
| Provide information to the caregiver about the patient/client’s vision loss and prognosis | ⭘ | ⭘ |
| Signpost caregiver to other sources of help (including sight loss and carer support charities) | ⭘ | ⭘ |
| Provide advice to caregivers on supporting the patient/client’s eye health (e.g. medication management, applying drops) | ⭘ | ⭘ |
| Refer caregiver to social services | ⭘ | ⭘ |
| Provide advice to caregivers on looking after their own physical and/or mental health | ⭘ | ⭘ |
| Discuss aids or strategies that may reduce caregiver burden/effort with daily activities (e.g. low vision aids, other assistive technologies, home adaptations) | ⭘ | ⭘ |
| Discuss the emotional aspects of caregiving | ⭘ | ⭘ |
| Provide counselling (or other formal psychological therapy) to caregiver | ⭘ | ⭘ |
| Provide advice to caregiver about housing, transport, education, benefits and/or employment | ⭘ | ⭘ |

1. **Please provide the name of organisation(s) where you have previously referred caregivers for support (if any):**
2. **Are you generally confident that caregivers have timely access to the support you recommend?**

Always

Frequently

Sometimes

Never/almost never

Don’t know

1. **Have you ever raised a safeguarding alert out of concern for the caregiver of a service user?**

Yes

No

1. **Do you feel that the overall quality of the support system for caregivers of your service users is generally…**

Excellent

Good

Adequate

Poor

Don’t know

1. **If more information/training regarding caregiver support was available to professionals, would you be interested?**

Yes, but too busy

Yes

No, I feel I know enough already

Not interested/not relevant to me

If you wish to and have time, please share your thoughts on any of the following questions regarding your experience working with caregivers. **You can leave these fields blank and skip to the end of the survey if you prefer.**

1. **In your view, what makes caring for someone with a visual impairment different to caring for someone with another chronic health condition?**
2. **What do you perceive to be the most serious gap or shortcoming in the provision of support for the caregivers of people with a visual impairment?**
3. **What kind of additional information/training/resources to better support the caregivers of your service users would be useful to you (if any)?:**
4. **If you have any other thoughts about improving support for caregivers, please briefly note them here:**

**Would you like to be entered into our prize draw? (If YES, we will need your contact details.)**

YES / NO

**Would you like to be informed of the study results? (If YES, we will need your contact details.)**

YES / NO

**If you have answered YES to either of the questions above**, please provide the following details:

Name: _______________________________

Telephone number: _______________________________

Email address: _______________________________

*We will only use these details to contact you for the purpose you have indicated – your details will not be used for anything else, and we will not pass them on.*

1. More information about Charles Bonnet Syndrome can be found on the Esme’s Umbrella website: <https://www.charlesbonnetsyndrome.uk/> [↑](#footnote-ref-1)
2. See <https://www.england.nhs.uk/wp-content/uploads/2016/05/identifying-assessing-carer-hlth-wellbeing.pdf> Paragraph 3.3.7, for official definition of vulnerable carer groups [↑](#footnote-ref-2)
